# When can we trust structural models derived from pair distribution function measurements?

**DOI:** 10.1039/d4fd00106k

**Published:** 2024-05-30

**Authors:** Phillip M. Maffettone, William J. K. Fletcher, Thomas C. Nicholas, Volker L. Deringer, Jane R. Allison, Lorna J. Smith, Andrew L. Goodwin

**Affiliations:** a Department of Chemistry, University of Oxford, Inorganic Chemistry Laboratory South Parks Road Oxford OX1 3QR UK andrew.goodwin@chem.ox.ac.uk; b School of Biological Sciences, University of Auckland 1142 Auckland New Zealand

## Abstract

The pair distribution function (PDF) is an important metric for characterising structure in complex materials, but it is well known that meaningfully different structural models can sometimes give rise to equivalent PDFs. In this paper, we discuss the use of model likelihoods as a general approach for discriminating between such homometric structure solutions. Drawing on two main case studies—one concerning the structure of a small peptide and the other amorphous calcium carbonate—we show how consideration of model likelihood can help drive robust structure solution, even in cases where the PDF is particularly information-poor. The obvious thread of these individual case studies is the potential role for machine-learning approaches to help guide structure determination from the PDF, and our paper finishes with some forward-looking discussion along these lines.

## Abstract 2

In this paper, we explore the importance of biasing structure refinement by model likelihood as a methodology for discriminating between different possible structure solutions.

## Graphical abstract

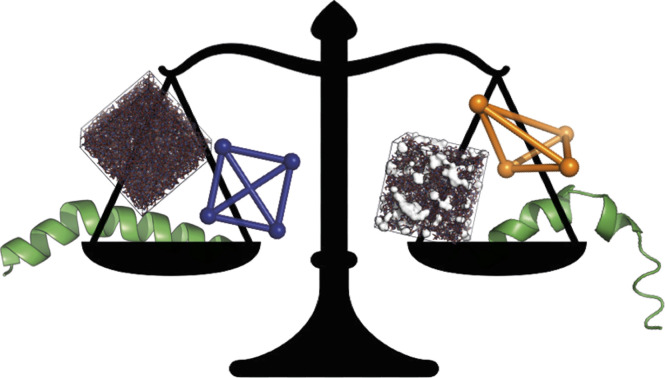

## 1 Introduction

The pair distribution function (PDF) is a well-known and widely-used measure of local structure in complex materials.^1–4^ Essentially a (weighted) histogram of interatomic distances, it is an appealing metric for a number of reasons. First, it is well-defined for any material—whether crystalline or amorphous, molecular or extended, liquid or solid. One can always meaningfully ask how likely it is to find a pair of atoms separated by a given distance. Second, the PDF can be can be measured experimentally as the Fourier transform of the X-ray, neutron, or electron total scattering functions—all that differs in each case is the weighting of contributions to the PDF from different atomic scattering strengths. And, third, the PDF can be calculated directly from the coordinates of any structural model, and hence it is straightforward to test consistency between model and experiment by comparing calculated and observed PDFs.

The inverse problem—namely using PDF measurements to determine structure—is a famously difficult but important challenge,^5,6^ framed back in 2007 as the ‘nanostructure problem’.^7^ In a landmark study of the day, Billinge and co-workers showed that the PDFs of small highly-symmetrical molecules such as C_60_ appeared to contain sufficient information to constrain fully the corresponding three-dimensional structures.^8^ The problem of structure solution then became, in this paradigm, one of developing intelligent algorithms for exploring configurational space to identify the unique configuration capable of reproducing a given PDF.

**Fig. 1:**
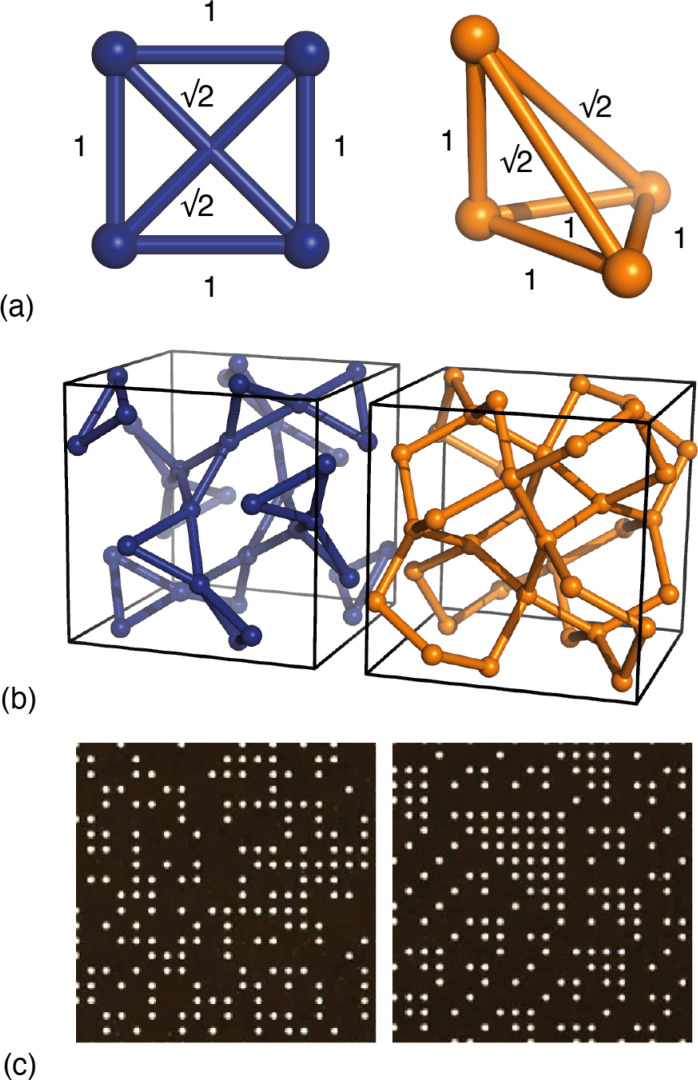
Examples of homometry. (a) Two arrangements of four atoms that give rise to the same set of six pairwise distances. The apex atom of the pyramidal arrangement of the right-hand side sits directly above one vertex of the basal equilateral triangle. (b) In the space group *Ia*3̄, inversion of the *x*-coordinate for atoms on the 24d Wyckoff site (*x*,0,¼) generates a pair of structures that are neither superimposable nor mirror-images of one another.^14^ The two structures contain the same interatomic vectors (up to inversion) and hence give rise to identical Bragg intensities.^15^ (c) Examples of disordered site-occupancy models that share identical pair correlations but different three-body correlations. The two images are fragments of much larger configurations that give rise to identical diffuse scattering patterns. This panel is adapted from ref. 18 with permission from the International Union of Crystallography.

Yet it has long been known that this apparent success cannot be universal, since meaningfully-different structural models can give rise to identical PDFs.^9,10^ The simplest example of which we are aware concerns the square and pyramidal arrangements of four atoms shown in Fig. 1(a). For specific geometries, the two arrangements contain precisely the same set of six interatomic distances, and so the corresponding PDFs are, by definition, equivalent. In general, as the number of atoms in a system grows, so too does the number of possible equivalent geometries. It is no surprise then that uniqueness is a particular challenge for amorphous materials, with a-Si being the canonical example.^9–13^ Even fully-ordered crystals are not immune to these limitations, and a useful historical observation in this regard is Pauling’s identification of what Patterson called ‘homometry’ in systems of particular crystal symmetries [Fig. 1(b)].^14,15^ A subtler, but more broadly relevant, example is the insensitivity of diffraction measurements to discriminating enantiomers of chiral crystals in the absence of anomalous diffraction effects. Three-dimensional PDF measurements^16,17^—in which the 3D diffuse scattering function of a disordered crystal is transformed to give a direction-dependent PDF as a scalar field *g*(**r**)—are certainly more information-rich than the conventional (one-dimensional) PDF, but again it is known that meaningfully different disordered states can give identical diffuse scattering patterns (and hence *g*(**r**) functions) when the differences involve multi-body correlations beyond second order [Fig. 1(c)].^18^

Mindful of this ambiguity, we argue here that the real problem to be addressed is not “given a PDF, what is the corresponding structure?”—since in general there is no unique solution—but rather “given a PDF, what is the most likely corresponding structure?”. Casting this point in the language of Bayes,^19^ one might compare the likelihoods *P* of two models A and B, given a PDF, as1
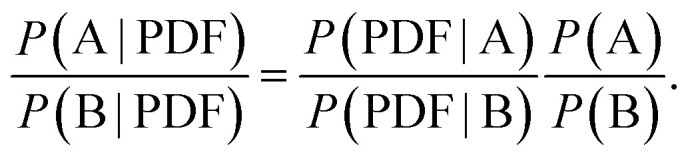
In other words, the relative abilities of models A or B to account for the PDF (*i.e.*, the first ratio on the right-hand side of eqn (1)) ought to be weighted by the corresponding *a priori* likelihoods of the models themselves. The implication is that, in cases where model likelihoods are sufficiently well characterised or calculable, any ambiguity of fitting to the PDF might nonetheless be ameliorated (and perhaps even overcome entirely).^20^

In this paper, we explore two particular directions in which this likelihood-weighted approach to interpreting the PDF might be developed. The first makes use of likelihoods determined using statistical analysis of large databases. We use a test case based on protein structure (for which statistical analysis is particularly mature), showing that surprisingly detailed structural information can be extracted from an ostensibly featureless protein PDF by exploiting backbone conformational analysis. The second example uses state-of-the-art empirical potentials to compare the energetics of competing atomistic models, which can in turn be interpreted as relative likelihoods through the Boltzmann formalism. The specific case we consider is that of amorphous calcium carbonate, with our analysis drawing heavily on the results of ref. 21 and 22. The common thread of these case studies is the potential for applied statistics and machine-learning approaches to help guide structure determination from the PDF. We conclude with a forward-looking discussion along these lines.

## 2 Results

### 2.1 Pathology of unbiased fitting to the PDF

Before launching into our case studies, we begin by making a general point regarding the counterintuitive statistical bias against simplicity when fitting to the PDF. Since the PDF represents a histogram of distances, the process of structure solution is essentially one of assigning elements in a distance matrix. Two solutions are equivalent if there exists a permutation of atom labels that transforms the corresponding distance matrices, one into the other. In the case of the six distances of the four-atom models shown in Fig. 1(a), for example, it can be shown that there are exactly two distinct solutions, given by matrices of the form2
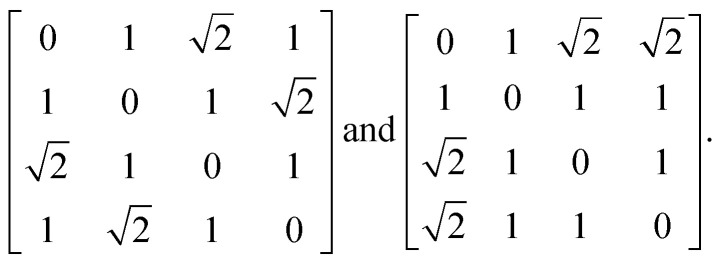
Here, each element *d*_*ij*_ gives the distance between atoms *i* and *j*. Note that there is no permutation of labels that would transform the two matrices in eqn (2) into one another. The 
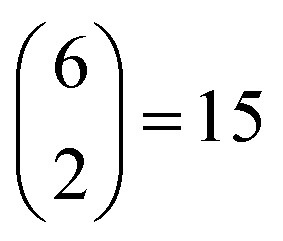
 possible ways of assigning the set of distances 
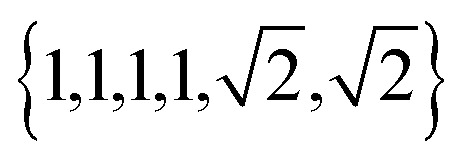
 amongst pairs of four atoms correspond to 3 distance matrices of the first kind (*i.e.*, the square) and 12 of the second kind (the triangular pyramid). Hence, a purely stochastic approach is four times more likely to obtain the second solution than the first, despite the two corresponding to precisely the same PDF.

This bias has as its origin the different symmetries of the two solutions: there are simply more unique ways of labelling the atoms in a lower-symmetry solution than in a higher-symmetry one. Generalising beyond the toy model of Fig. 1(a), the implication is that stochastic approaches are statistically biased towards structure solutions with maximal variance amongst atom environments. Nature, as Pauling would have it, seems to be biased in precisely the opposite sense: from a purely empirical perspective, one finds that structures tend to adopt as few different environments as possible (‘law of parsimony’).^23^ And so the unguided structure solution from the PDF suffers not only from the often-discussed uniqueness problem, but from a subtler pathology whereby the most natural structure solutions are also inherently the least likely to be found.

We encountered this problem, albeit in reverse, many years ago when seeking to improve the effectiveness of Reverse Monte Carlo (RMC) approaches to nanostructure solution. In its purest form, RMC is a good example of an unguided stochastic methodology for structure solution. It uses the Metropolis algorithm^24^ to accept or reject proposed moves within a randomly-arranged box of atoms, with the acceptance criterion dependent on the fit between calculated and observed PDF.^5^ After many successive moves, and once equilibrium is established, RMC gives a structure that is statistically biased towards maximally-variant solutions, as described above. Within the field, one often uses phrasing along the lines of ‘the most disordered structure consistent with the data’.^25^ By including an additional term within the Metropolis acceptance criterion that penalised variance, we were able to guide structure solution (in favourable cases) to more natural solutions. In the formalism of eqn (1), this so-called INVERT approach effectively interpreted the prior probability of a model in terms of the variance of atom environments within that model.^10,26^

### 2.2 Exploiting statistical knowledge

There are many cases where our understanding of prior likelihood is much more robust than inference on the basis of atom-environment variance alone. Pauling’s law of parsimony was developed through his own empirical observations of the many thousands of structures he encountered (and is not universal^27^). However, we now have access to vastly superior statistical information from the enormous number of structures that have since been solved. This places us in a position to determine accurate estimates of the likelihood of a particular structural model, based on its similarity or difference from other known structures.

Nowhere is this possibility more effectively applied than in the field of sequence-driven protein structure prediction.^28^ Algorithms such as AlphaFold^29,30^ exploit the enormous volume of structural information contained within the protein data bank (PDB) to predict a protein fold (and the corresponding likelihood) from knowledge of amino-acid sequence alone. Consequently we sought to establish whether sequence-derived prior probabilities might be able to guide the structure solution of small proteins from their corresponding PDFs.

**Fig. 2:**
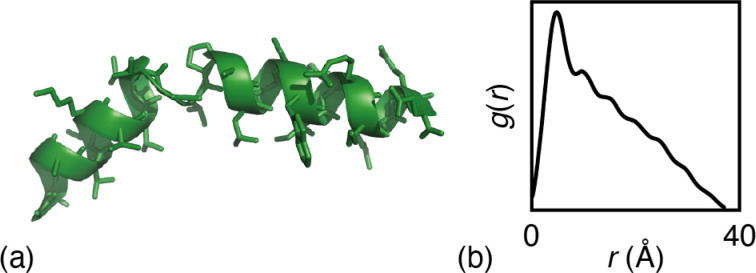
(a) Representation of the structure of melittin: the peptide forms an alpha helix with an off-center bend. (b) The simulated PDF of melittin.

**Fig. 3:**
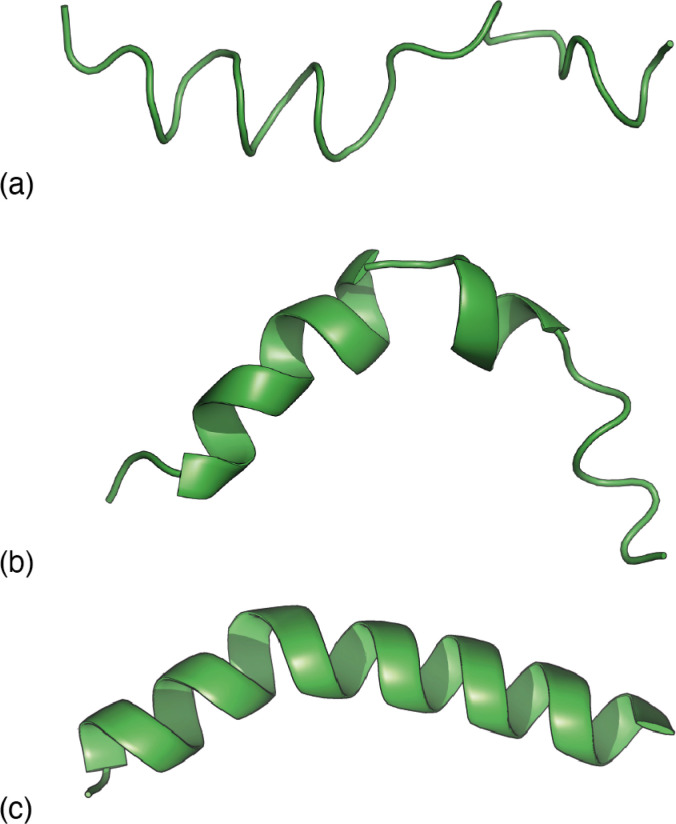
Representative melittin structure ‘solutions’ obtained by RMC fitting to (a) the PDF alone, (b) the TALOS-N likelihoods alone, and (c) the PDF when biased by TALOS-N likelihoods.

The particular example we have explored as a proof of principle is the case of the small (27-residue) peptide melittin, a key component of bee venom [Fig. 2(a)].^31^ Its corresponding PDF, which might be measured experimentally in suitable small-angle scattering experiments, is essentially featureless [Fig. 2(b)], but nonetheless contains basic information concerning the size and shape of the protein.^32^ The degree of information content within the PDF was interrogated using a simple RMC approach as follows. We first generated a model of melittin from its known amino-acid sequence, but with random backbone torsion angles. These angles were then treated as RMC variables: each successive move involved changing an individual torsion angle by some small random amount, and the move was accepted or rejected using the usual Metropolis algorithm applied to the quality of fit to the PDF. For ease of calculation, our implementation used the cumulative PDF 
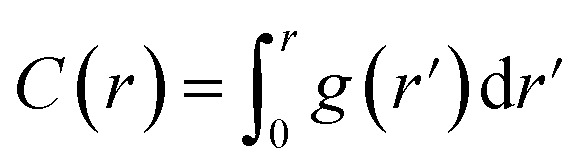
 derived simply from the alpha carbon positions;^10,33^ here *g*(*r*) denotes the relative probability of two alpha carbons being separated by a distance *r*. A typical converged structure ‘solution’ is represented in Fig. 3(a). As anticipated, the basic envelope of the protein is correctly modelled, but details of the protein fold are lost.

Our next step was to incorporate information regarding the likelihood of a given model of melittin structure. There are a variety of approaches one might take, but the one on which we focussed was to exploit the statistical information regarding the likelihood of individual residue torsion angles provided by the code TALOS-N.^34,35^ Taking the known melittin structure as a reference, we first computed the corresponding backbone NMR chemical shifts using the SPARTA+ code.^36^ TALOS-N, which is trained on the same dataset as SPARTA+, then returns, for each residue, a prior likelihood *P*(*ϕ*, *ψ*) of it adopting a conformation with torsion angles *ϕ*, *ψ* given the corresponding NMR chemical shifts.^34,35^ By design, these likelihoods include the statistical information contained within Ramachandran distributions.^37,38^ Here we are intentionally using a prior calculation process that is less powerful than state-of-the-art structure prediction protocols in order to demonstrate the complementary roles of the two terms in eqn (1). So, for example, a model of melittin generated using the TALOS-N-likelihoods alone gives a solution that is not fully correct [Fig. 3(b)].

Combining these two strands, we then carried out a new RMC refinement against the melittin PDF in which the selection of torsion angles was biased by the prior likelihoods given by TALOS-N. The result can be interpreted as the statistically most likely structure solution that fits the PDF. A typical solution we obtained is shown in Fig. 3(c). What is clear is that it matches more closely the known structure than solutions obtained using either the PDF data or model likelihoods by themselves. Quantifying this point, the root mean-squared deviation between the refined and known structure is less than 2 Å for the biased RMC result, and significantly larger than this value (as much as 8 Å) for both unbiased RMC and TALOS-N-alone refinements.

**Fig. 4:**
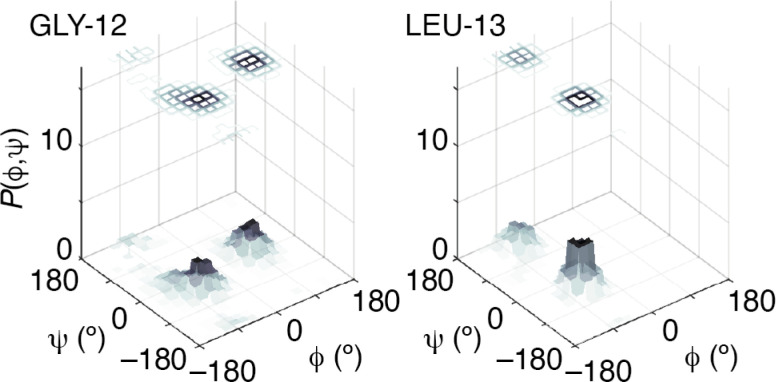
Representative prior probability distributions of backbone torsion angles determined for melittin using TALOS-N for two residues. Note the presence of a small number of likely conformations in each case.

The success of this combined approach is easily rationalised. It is often the case that, for individual residues, there are a handful of maxima in the torsional likelihood distributions; some examples are shown in Fig. 4. Choosing one or the other of two similarly-likely conformations can have a relatively stark effect on the overall protein fold, since the collective conformation propagates from residue to residue. This is why the information within the PDF regarding overall shape—as coarse at it is—is useful nonetheless in selecting which particular conformations are observed in practice.

Of course, we make no claim that this biased RMC approach is a general methodology for protein structure solution from PDF data. Our study is simply one of proof-of-principle to demonstrate the (perhaps surprising) power of incorporating statistical likelihoods to bias PDF-driven structure determination, as envisaged by eqn (1).

### 2.3 Exploiting energetics

An alternative approach for comparing the likelihoods of two competing models, where their corresponding energies are known, is to consider the Boltzmann factor3
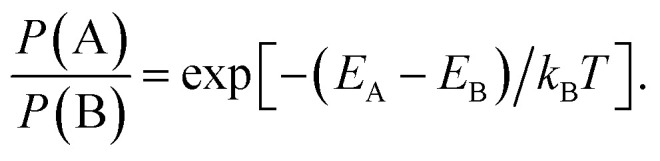
Clearly the ability of this term to meaningfully discriminate models through eqn (1) depends on the accuracy with which energies can be determined. Many simple constraints and restraints conventionally applied during RMC refinements can be recast in terms of simple effective potentials. For example, a closest-approach constraint that forbids atoms from approaching nearer than a critical value *r*_c_ might be understood in terms of a potential4
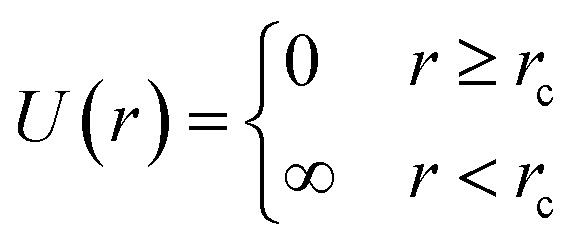
contributing to a lattice energy 
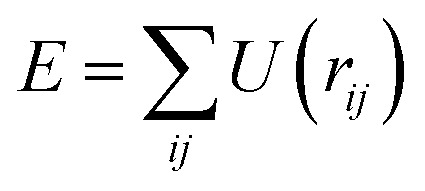
.^5^ In this picture, any two models that satisfy the closest-approach constraint have the same (zero) energy and are treated as equally likely; a model that violates the constraint has infinite energy and hence a likelihood of zero. Likewise, bond-angle and bond-length restraints—which are routinely used in RMC refinements—behave as simple empirical potentials that weight the solution space accordingly.^39,40^

One chemical system for which very-high-quality empirical potentials have been developed is that of calcium-carbonate–water. The state-of-the-art potential includes electrostatic terms, rigid carbonate ions, and a combination of Buckingham and Lennard-Jones two-body potentials; its quality is seen in the ability to reproduce a variety of key thermodynamic properties, including the calcite–aragonite phase transition and the calcite dissolution enthalpy.^41,42^ This potential is significantly more sophisticated than the conventional harmonic restraints used in many RMC refinements, and so might be expected to provide a particularly robust measure of the likelihood of competing candidate models.

**Fig. 5:**
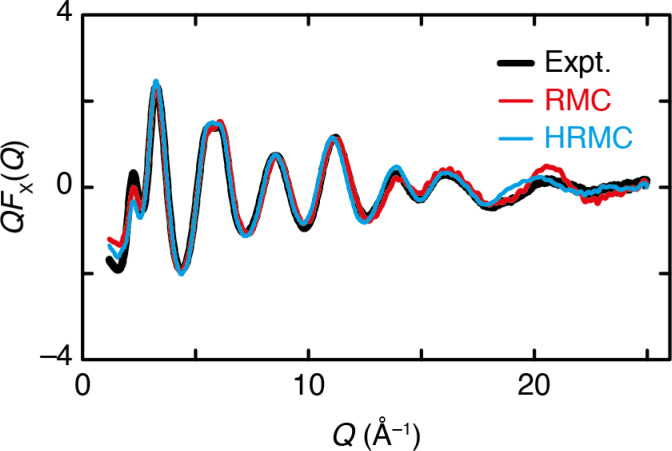
The *Q*-weighted X-ray total scattering function *QF*_X_(*Q*) of ACC (black line) can be accounted for satisfactorily using both RMC and HRMC refinement strategies (red and blue lines, respectively).

We have performed two separate RMC studies of amorphous calcium carbonate (ACC), both driven by the same X-ray total scattering (*i.e.*, PDF) data. The first study, carried out in 2010, made use of simple closest-approach constraints of the type described by eqn (4);^21^ the second, much more recent, study included the state-of-the-art calcium-carbonate–water potential of ref. 22 and 41. The inclusion of such sophisticated empirical potentials within an RMC refinement process is usually referred to as a ‘hybrid’ RMC (or HRMC) methodology because it is essentially intermediate between force-field simulation and data-driven refinement.^43^ A comparison of the two corresponding fits to data is given in Fig. 5. While there are subtle differences between the two—most noticeably at very low-*Q* and again around a weak oscillation near *Q* ≃ 20 Å^−1^—the fits are comparable in quality. This point can be quantified by comparing the fit metrics5
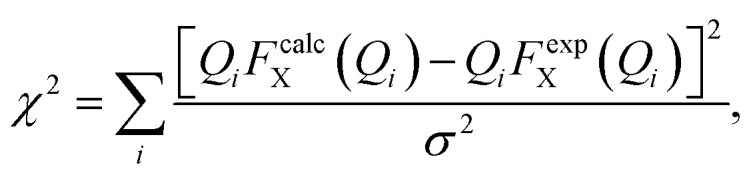
where the sum is taken over data points *i* and *σ* is the estimated uncertainty on the experimental *Q*-weighted X-ray total scattering *QF*_X_(*Q*) values. Here, *Q* is the magnitude of the scattering vector, and the superscripts ‘calc’ and ‘exp’ denote calculated and experimental values, respectively. We obtain *χ*^2^ = 13.7 and 17.5 for RMC and HRMC methodologies, respectively. To put this difference in perspective, a fit obtained using the empirical potentials alone gave *χ*^2^ = 71.2.

**Fig. 6:**
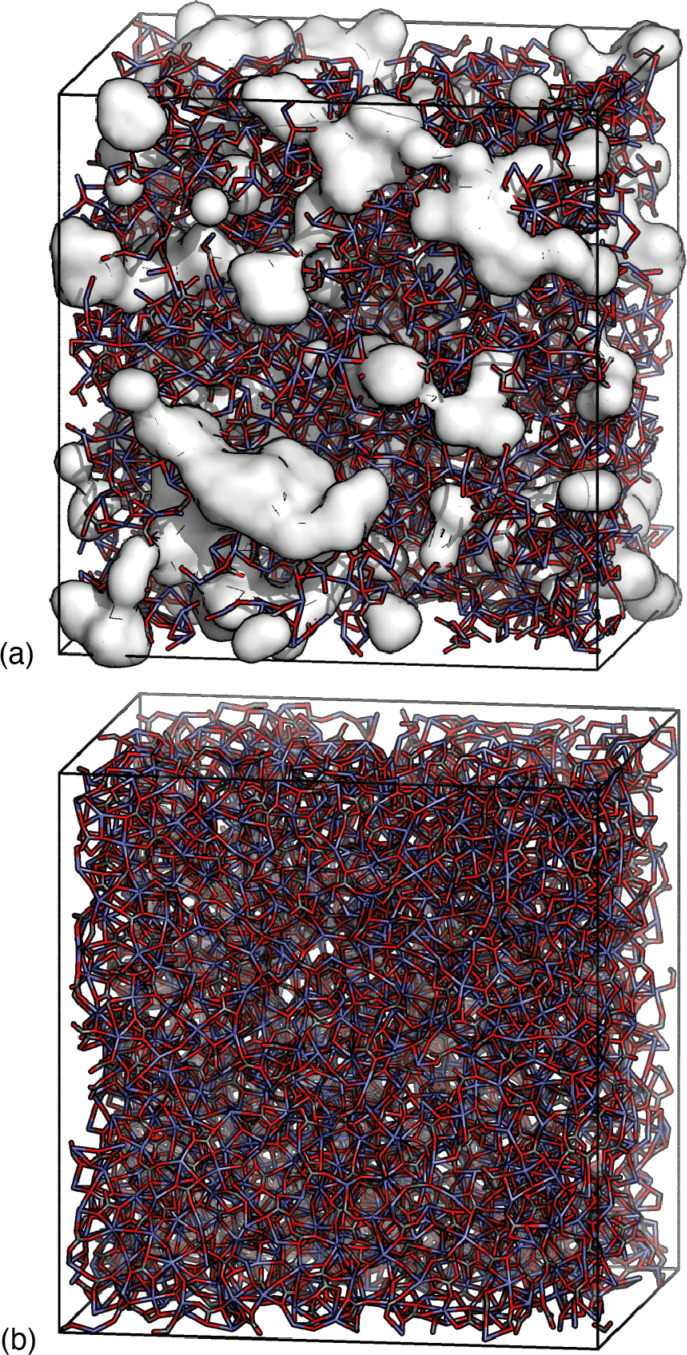
Comparison of ACC models determined using (a) RMC and (b) HRMC refinement strategies. In both cases, only the connected calcium-carbonate network is shown in stick representation. The presence of Ca-poor regions in (a) is highlighted by the white surfaces, which correspond to regions of the configuration more than 4 Å away from any Ca atom. There are no such regions in the HRMC model.

**Fig. 7:**
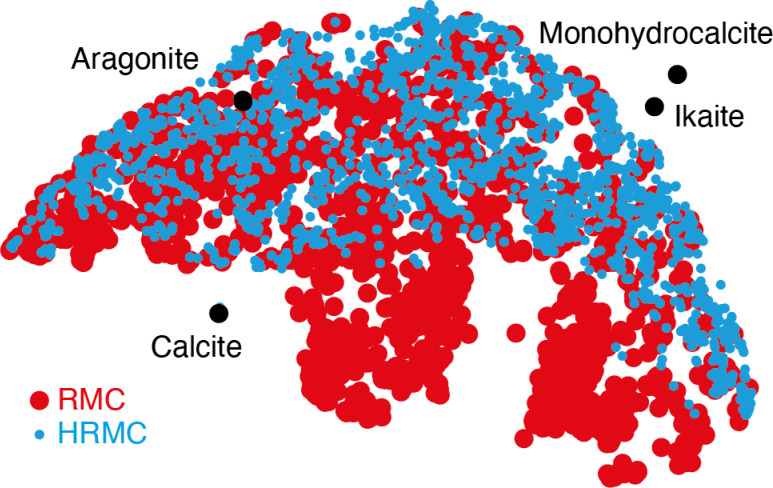
Two-dimensional embedding representing the distribution of local Ca-atom arrangements in RMC and HRMC models of ACC and also some key crystalline polymorphs of (hydrated) calcium carbonates. In this embedding, Ca atoms whose local environments are similar appear as points close to one another.

Despite this ostensible similarity in fit-to-data, the underlying configurations from which the fits were calculated are remarkably different. Perhaps the key distinction is that Ca-ion distributions are heterogeneously distributed in the RMC configuration but are essentially uniform in the HRMC configuration [Fig. 6]. This is an important difference, because the presence of Ca-poor channels had been interpreted as a possible mechanism for intercalation-driven stabilisation of ACC.^21^ The degree of heterogeneity in Ca arrangements for the two models can be compared quantitatively using a smooth overlap of atomic positions (SOAP) similarity function.^44^ For each Ca atom in each configuration, its local environment of neighbouring Ca atoms within a given radius (up to 4.5 Å) was expressed in terms of a power spectrum **p** that contains coefficients of a neighbour-density expansion into a local basis with radial and spherical harmonic terms. The similarities between all Ca environments can then be visualised by performing dimensionality reduction on these vectors, here giving a two-dimensional embedding (or map) using UMAP.^45,46^ In this representation, atoms in a similar environment appear near to one another. The UMAP maps for both the RMC and HRMC configurations are shown in Fig. 7, which also includes Ca environments in key crystalline polymorphs. Our point regarding the greater variance in Ca environments in the RMC configuration relative to HRMC is made clear by the different areas covered by the corresponding data points in this map.

The potential of ref. 41 also gives an enormous energy difference between the two configurations: we calculated the RMC configuration to be approximately 880 kJ per mole of CaCO_3_·H_2_O less stable than the HRMC configuration.^22^ The number is particularly large because of the variation in charge distribution between the two configurations. Interpreted in the context of the Boltzmann factor, this energy difference translates to a vanishingly small probability that the RMC result can be correct.

So the key points of our comparative study are that (i) remarkably different models of ACC can result in very similar fits to data, and (ii) high-quality empirical potentials allow discrimination between these models *via* the formalism of eqn (1).

## 3 Discussion

Turning to the question raised in the title of this paper, these various small studies suggest some conditions under which we might hope to trust structural models derived from fitting to PDF data. We argue there are two scenarios. The first is the case where PDF data are sufficiently information-rich that high-quality fits are possible only for correct structural models. Here the ratio *P*(PDF|A)/*P*(PDF|B) is the term in eqn (1) that is responsible for discriminating correct from incorrect solutions. Disordered crystals (rather than amorphous materials) and the use of single-crystal measurements (*e.g.*, 3D-ΔPDF) will favour this scenario. The second scenario corresponds to cases where we have a suitably robust measure of the relative likelihoods of competing models. Here it is the *P*(A)/*P*(B) term in eqn (1) that discriminates. We have shown how likelihood might be introduced either through statistical sampling (as in the case of torsion-angle-probabilities for peptide conformations) or through energetics *via* the Boltzmann factor (as in the case of ACC).

In both respects, one might expect machine-learning (ML) approaches to play an increasingly important role. On the one hand, libraries of previously solved PDF data—with each observed pattern “labelled” with the corresponding structure—can be used to train supervised ML models to assign the structure that corresponds to an unknown PDF, and unsupervised ML may help to analyse large and complex experimental datasets; see ref. 47 and references therein. And, on the other hand, ML methods are now firmly established for fitting accurate interatomic potential models based on quantum-mechanical reference data,^48,49^ and such machine-learned potentials may very well facilitate energy calculations for guiding robust HRMC refinements. In the case of ACC, we were fortunate to have access to high-quality empirical potentials; however, there are many chemical systems (not least amorphous silica^50^) for which even the best empirical potentials available face challenges in capturing the subtleties of the potential-energy landscape. As ML potentials become available for systems of ever-increasing complexity (*e.g.* metal–organic frameworks^51^), we anticipate that HRMC approaches will become increasingly popular and effective in determining high-quality structure models informed at once by both experiment and computation.

On a purely qualitative level, eqn (1) also provides a useful heuristic for assessing competing models, even in cases where quantitative measures of model likelihoods are inaccessible. Experience in the field often gives one a sense for which structure solutions are more or less likely to be chemically and/or physically reasonable, and this intuition (for want of a better word) may itself be useful in discriminating between models with similar fits to data. An example from our own experience is that of characterising the orbitally-disordered (high-temperature) phase of LaMnO_3_.^52^ We found that three different models with very different underlying physics were almost equally able to reproduce a combination of neutron and X-ray PDF measurements. A key difficulty was that, at the high temperatures involved, the PDF peak widths were relatively large and as a consequence it was very difficult to disentangle static and dynamic contributions to local distortions. In such cases, we argued that the physicality of the models themselves ought to be taken into account, and this influenced our own conclusions regarding the nature of the high-temperature phase of LaMnO_3_ (rejecting, in that case, the model that emerged from RMC refinements).

## 4 Concluding remarks

If there is one general conclusion to be drawn from our study, it is to underscore the point that the ability to match an experimental PDF is a necessary but not sufficient condition in validating a candidate structural model. We have shown that unconstrained fitting to the PDF is actually biased against naturally-preferred solutions, but that by weighting fits according to model likelihood, it is possible to overcome this bias and correctly discriminate between homometric models. Ultimately, our conclusions here with respect to interpretation of scattering data are converging on the same central tenets of the field of NMR crystallography:^53^ namely, that it is by combining the complementary sensitivities of different computational and experimental approaches that the task of structure ‘solution’ is most robustly addressed.

## Conflicts of interest

There are no conflicts to declare.
